# miR-362-3p acts as a tumor suppressor by targeting SERBP1 in ovarian cancer

**DOI:** 10.1186/s13048-020-00760-2

**Published:** 2021-02-01

**Authors:** Shujun Cao, Na Li, Xihong Liao

**Affiliations:** 1 Department of Obstetrics and Gynecology, Shanghai Songjiang District Central Hospital, 748, Zhongshan Middle Road, Songjiang District, Shanghai, China; 2 Department of Obstetrics and Gynecology, the First People’s Hospital of Chenzhou, Southern Medical University, Chenzhou, China

**Keywords:** Ovarian cancer, miR-362-3p, SERBP1, Molecular mechanisms, Metastasis

## Abstract

**Background:**

Ovarian cancer is the leading lethal gynecological cancer and is generally diagnosed during late-stage presentation. In addition, patients with ovarian cancer still face a low 5-year survival rate. Thus, innovative molecular targeting agents are required to overcome this disease. The present study aimed to explore the function of miR-362-3p and the underlying molecular mechanisms influencing ovarian cancer progression.

**Methods:**

The expression levels of miR-362-3p were determined using qRT-PCR. Gain-of-function and loss-of-function methods were used to detect the effects of miR-362-3p on cell proliferation, cell migration, and tumor metastasis in ovarian cancer. A luciferase reporter assay was performed to confirm the potential target of miR-362-3p, and a rescue experiment was employed to verify the effect of miR-362-3p on ovarian cancer by regulating its target gene.

**Results:**

miR-362-3p was significantly downregulated in ovarian cancer tissues and cell lines. In vitro, our data showed that miR-362-3p suppressed cell proliferation and migration. In vivo, miR-362-3p inhibited ovarian cancer growth and metastasis. Mechanistically, SERBP1 was identified as a direct target and functional effector of miR-362-3p in ovarian cancer. Moreover, SERBP1 overexpression rescued the biological function of miR-362-3p.

**Conclusions:**

Our data reveal that miR-362-3p has an inhibitory effect on ovarian cancer. miR-362-3p inhibits the development and progression of ovarian cancer by directly binding its target gene SERBP1.

Ovarian cancer is the leading lethal gynecological cancer and is generally diagnosed during late-stage presentation [1]. It has been estimated that ovarian cancer accounts for 2.5% of female cancer occurrences and 5% of cancer-related deaths [1]. Despite great advances in early detection and systematic therapies over the past few years, patients with ovarian cancer still face a low 5-year survival rate [2]. Thus, it is urgent that new potential therapeutic targets for ovarian cancer be identified to improve the survival rate.

MicroRNAs (miRNAs) are a class of small noncoding RNAs consisting of 20 to 22 nucleotides and play a pivotal role in tumor invasion and tumorigenicity by binding to the 3’ untranslated regions (3’ UTRs) of their target genes [3, 4]. Mounting evidence indicates that miRNAs have special functions in regulating cell migration, invasion, proliferation, and differentiation in various tumors [5–8]. Compared to normal tissues, ovarian cancer tissues have also shown differential expression of miRNAs [9, 10]. Research on miR-362, a recently discovered miRNA, has been shown to play an essential role in modulating a variety of physical activities and regulating the tumorigenicity and progression of several tumor types [11]. Further, miR-362-3p has been shown to inhibit cell proliferation and invasion in colorectal cancer, glioma, and cervical adenocarcinoma through various mechanisms [12, 13]. However, the roles and mechanisms of miR-362-3p in ovarian cancer progression are still unknown.

In the present study, we aimed to investigate the possible function of miR-362-3p in ovarian cancer. We detected lower miR-362-3p expression in ovarian cancer tissues and cells than in normal tissues and cells. Functional analysis indicated that miR-362-3p repressed cell proliferation, invasion, and migration in vitro and in vivo. Moreover, we identified that miR-362-3p repressed ovarian cancer proliferation by directly combining with and regulating SERPINE1 mRNA-binding protein 1 (SERBP1).

## Materials and methods

### Expression profiles of miRNAs

Using the Affymetrix Multispecies miRNA-2 Array (GPL14613), miRNA expression data of GSE47841 were obtained from the publicly available Gene Expression Omnibus (GEO) database (http://www.ncbi.nlm.nih.gov/geo/). The database included the disease-related expression data of miRNAs from 12 high-grade serous ovarian carcinomas (HSOCs) and 9 clear cell ovarian carcinomas (CCOCs) as cases and 9 ovarian surface epithelium (OSE) tissues as controls. The R package was used to calculate the false discovery rate (FDR) and whether the log2 fold change (log2 FC) was > 1. An adjusted *P* < 0.05 was considered significant. The limma and pheatmap packages were used to draw volcano plots and heatmaps, respectively.

### Cell culture

The ovarian cancer cell lines OVCAR3, CAOV3, HO-8910, and SKOV3 and the normal human ovarian epithelial cell line IOSE80 were obtained from the American Type Culture Collection (ATCC) (Manassas, VA, USA; https://www.atcc.org/). These cell lines were cultured in Dulbecco’s modification of Eagle’s medium Dulbecco (DMEM) supplemented with 10% fetal bovine serum (FBS) and 1% antibiotics in an incubator with 5% CO_2_ at 37 °C.

### Reagent and transfection

The anti-SERBP1 and anti-glyceraldehyde-3-phosphate dehydrogenase (GAPDH) primary antibodies were purchased from Abcam (Cambridge, MA, USA) and CST (Cell Signaling Technology, USA), respectively. The miR-362-3p mimic, miR-362-3p inhibitor, and negative control (NC) (scrambled sequence) were synthesized by and purchased from Shenggong (Shanghai, China). The sequences are presented in Table 1. All these molecules were transfected into cells using Lipofectamine 2000 (Invitrogen, USA) according to the manufacturer’s protocol.

**Table 1:** The sequence of miRNA

miRNA	Sequence
miR-362-3p	5’-AACACACCUAUUCAAGGAUUCA-3’
miR-362-3p mimics	5’-AACACACCUAUUCAAGGAUUCA-3’
miR-362-3p inhibitor	5’-UGAAUCCUUGAAUAGGUGUGUU-3’
miRNA NC (Scramble)	5’-UUCUCCGAACGUGUCACGUTT-3’

*NC*, negative control

### Dualluciferase reporter assay

Target genes of miR-362-3p were predicted by TargetScan (https://www.targetscan.org/), miRWalk (http://mirwalk.umm.uni-heidelberg.de/), and miRTarBase (http://mirtarbase.mbc.nctu.edu.tw/php/index.php). Only target genes predicted by all three databases were included in this study. A dual-luciferase reporter assay was used to assess the effects of miR-362-3p on the expression of the SERBP1 gene. The 3’ UTR of SERBP1 was amplified using PCR and cloned into a pGL3-promoter vector (Promega, Madison, USA). SKOV3 cells were cotransfected with the miR-362-3p mimic and pGL3-promoter-wtSERBP1 using the Lipofectamine 2000 Transfection Agent (Invitrogen, USA). The scrambled miRNA mimic was used as a control. The 3’ UTR mutant was a mutant form of the SERBP1 3’ UTR within the miR-362-3p binding site. After a transfection period of 48 h, the luciferase activity was analyzed using a Dual-luciferase Reporter Assay Kit (Promega, Madison, USA).

### Construction and transfection of SERBP1 overexpression vectors

The full-length open reading frame of SERBP1 (NM_001018067.2) was cloned and inserted into the expression vector pcDNA3.1(+). The primer pairs were SERBP1-F: 5’-CCCAAGCTTATGCCTGGGCACTTACAGG-3’ (Hind III) and SERBP1-R: 5’-CGGAATTCTTAAGCCAGAGCTGGGAATG-3’ (EcoRI). The resulting vectors are referred to as oeSERBP1. The empty vector pcDNA3.1(+) is referred to as the vector. All these vectors were transfected into cells using Lipofectamine 2000 (Invitrogen, USA) according to the manufacturer’s protocol.

### Real‐time RT-PCR

Total RNA from cells and tissues was extracted using TRIzol (Invitrogen, USA) according to the manufacturer’s directions. To quantify the mRNA levels, DNA-free RNA was reverse-transcribed using the M-MLV Reverse Transcription Kit (Promega). Quantitative reverse transcription-polymerase chain reaction (qRT-PCR) was carried out using Power SYBR® Green PCR Master Mix (Thermo Fisher). All reactions were repeated in triplicate wells. In this study, either GAPDH or human small nuclear RNA U6 was used for normalization. The 2^−ΔΔCt^ method was used for calculation. All the primer pairs are shown in Table 2.

**Table 2:** Primers used in the qPCR experiments

Primer name	Sequence
hsa-miR-362-3p-F	5’-GCGCGAACACACCTATTCAAG-3’
hsa-miR-362-3p-R	5’-AGTGCAGGGTCCGAGGTATT-3’
U6 small nuclear 1-F	5’-CTCGCTTCGGCAGCACA-3’
U6 small nuclear 1-R	5’-AACGCTTCACGAATTTGCGT-3’
SERBP1-F	5’-TAGACCGATTATTGACCGACC-3’
SERBP1-R	5’-TTGACAGTTCCCCAGTTGTG-3’
GAPDH-F	5’-AATCCCATCACCATCTTC-3’
GAPDH-R	5’-AGGCTGTTGTCATACTTC-3’

### Western blot

Radioimmunoprecipitation assay (RIPA) lysis buffer was used to extract the total proteins of the cells from all groups. After quantitating the protein concentration using a BCA protein assay (Thermo Fisher, USA), equal amounts of protein were separated using 10% sodium dodecyl sulfate-polyacrylamide gel electrophoresis (SDS-PAGE) and transferred onto PVDF membranes (Millipore, Billerica, USA). After blocking for 1 h at room temperature using 5% bovine serum albumin (BSA), the membranes were incubated with different primary antibodies at 4 °C overnight. After washing the membranes three times, they were incubated with the corresponding peroxidase-conjugated secondary antibodies for 2 h at room temperature. The ECL chemiluminescence system (Millipore, USA) was used to visualize the proteins. The relative protein expression level corresponds to the relative ratio of the integral optical density of the target protein to that of the reference protein GAPDH.

### Cell viability assay

At 12 h posttransfection, cells were plated at a density of 3 × 10^3^ cells/well in 96-well plates with 100 µl of culture medium as the blank control and cultured overnight at 37 °C. Cell viability was then determined by a CCK-8 assay (Dojindo, Japan) by adding 10 µl of CCK-8 reagent to each well and incubating the cells for 1 h. The absorbance of the plates was measured at 450 nm using a microplate reader at 0, 24, 48, and 72 h.

### Wound‐healing assay

SKOV3 and HO-8910 cells were trypsinized and seeded at a density of 8 × 10^5^ cells/well in 6-well plates. When the cells were 90% confluent, they were scraped with a 10 µl sterile pipette tip assisted by a ruler. After lightly washing the floating cells with PBS, they were cultured in serum-free medium with different interventions for 24 h. Images were captured at 0, 12, and 24 h using an inverted microscope (Olympus, Japan). The mean distance of cell migration was measured and analyzed by ImageJ (http://imagej.nih.gov/ij/; provided in the public domain by the National Institutes of Health, Bethesda, MD, USA). Each experiment was independently repeated three times.

### Cell cycle assay

SKOV3 and HO-8910 cells were trypsinized and seeded at a density of 3 × 10^5^ cells/well in 6-well plates. SKOV3 cells were transfected with the miR-362-3p mimic, and HO-8910 cells were transfected with the miR-362-3p inhibitor or the NC. Cells were harvested, washed, and fixed in ethanol at -20 °C for 12 h. Then, the collected cells were washed twice with PBS and treated with RNase A at 37 °C for 30 min while protected from light. Propidium iodide (PI) was then added for DNA staining. The distribution of cells with differing DNA contents was analyzed on a CytoFLEX flow cytometer (Beckman Coulter, USA) using FlowJo software (Tree Star).

### Transwell migration assay

A Transwell migration assay was performed to detect the cellular migration ability. Cells from all groups described in this study were seeded at 3 × 105 cells in the top of the chamber (Corning, Inc., USA) in DMEM without FBS. The lower chamber was filled with 750 µl of culture medium containing 10% FBS as a chemoattractant. After 24 h of incubation, cells that had migrated to the lower surface of the membrane were fixed in 4% paraformaldehyde for 20 min and then stained with crystal violet for 30 min. After washing with PBS three times, ten fields were randomly selected to be photographed, and the number of migratory cells was counted. Each experiment was independently repeated three times.

### In vivo xenograft model

The present study was performed according to the recommendations of the Guidelines for the Care and Use of Laboratory Animals. The protocol of animal experiments was approved by the research ethics committee of Shanghai Songjiang District Central Hospital, Shanghai, China. Each BALB/c nude mouse was subcutaneously injected with 5 × 10^6^ SKOV3 cells in 100 µl culture medium. On the 7th day after the injection when the mean tumor size was approximately 6 mm^3^, the mimic (mimic group, *n* = 6) and scrambled sequence (NC group, *n* = 6) were randomly injected via the tail vein (0.5 OD/day) for three weeks. The tumor size was measured by using a caliper every 3 days and was calculated by using the following formula: Volume = width^2^ × length × 0.5. All mice were sacrificed, and the tumor tissues were then harvested for further analysis after 33 days of cell inoculation.

### Lung metastasis mouse model

Twelve BALB/c nude mice were intravenously injected in the tail with 1 × 10^7^ cells in 100 µl culture medium. Then, they were randomly divided into a mimic group (*n* = 6) and an NC group (*n* = 6) and were injected with the mimic or scrambled sequence via the tail vein (0.5 OD/day) for three weeks. All mice were sacrificed 6 weeks after the injection, and the lung tissues were harvested for further analysis.

### Immunohistochemistry

The tissues obtained from the lungs of each group were sliced into 4 µm sections, rinsed with PBS, and fixed in 4% paraformaldehyde for 15 min. After rinsing again in PBS, the slides were blocked with 1% BSA and incubated with the primary antibody overnight at 4 °C. After several rinses, the slides were incubated with the corresponding fluorescent secondary antibody and 4,6-diamidino-2-phenylindole (DAPI) at room temperature for approximately 2 h. The slides were then visualized using a fluorescence microscope (Nikon, Japan).

### Hematoxylin and eosin staining

The lung tissues obtained from each group were paraffin-embedded and sliced into 4 µm sections. Then, the slides were heated in an incubator at 65 °C for 1 h, deparaffinized in xylene, rehydrated by graded ethanol, and stained with HE.

### Statistical analysis

Data in this study are presented as the means ± SDs. Each experiment was performed in triplicate. Differences between the two groups were determined using an independent sample *t* test. Differences between multiple groups were determined using two-way analysis of variance (ANOVA). All statistical analyses were performed using SPSS for Windows (Version 23.0; SPSS, Chicago, IL), with two-tailed *P* < 0.05 considered significant.

## Results

### miR-362-3p is downregulated in ovarian cancer tissues and cell lines

**Fig. 1:**
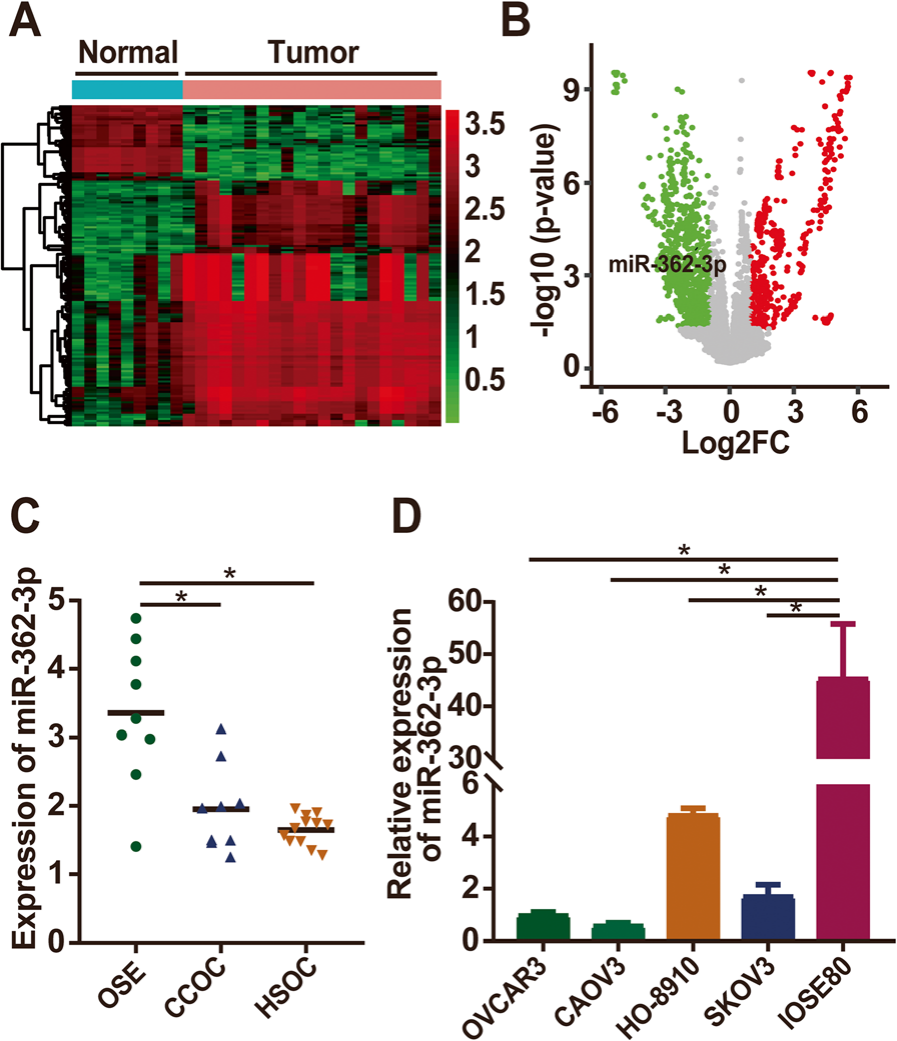
miR-362-3p is downregulated in human ovarian cancer. **a** Heatmap showing the hierarchical clustering of dysregulated miRNAs in ovarian cancer compared to those in normal control tissues in GSE47841 datasets. **b** Volcano plot showing the differential expression of miRNAs between ovarian cancer tissues and normal control tissues. **c** qPCR analysis of miR-362-3p expression in high-grade serous ovarian carcinoma (HSOC), clear cell ovarian carcinoma (CCOC), and ovarian surface epithelium (OSE) tissues. **d** qPCR analysis of miR-362-3p expression in ovarian cancer cell lines and normal ovarian epithelium cell lines. Data are shown as the means ± SDs. *P < 0.05

To assess the expression of ovarian cancer-related miRNAs that may have a role in ovarian cancer tumorigenicity, the expression data of the disease-related miRNAs from the GEO database (GSE47841) were analyzed first. The findings of this study demonstrated that miR-362-3p in ovarian cancer tissues was significantly downregulated compared to that in normal tissues (Fig. 1a, b). The expression of miR-362-3p was significantly higher in normal OSE tissues than in HSOC and CCOC tissues (Fig. 1c). Next, we detected miR-362-3p expression in normal ovarian epithelium cell lines (IOSE80) and ovarian cancer cell lines (OVCAR3, CAOV3, HO-8910, and SKOV3) and noticed that the expression of miR-362-3p was significantly higher in IOSE80 cells than in all ovarian cancer cell lines tested (Fig. 1d). Among the four ovarian cancer cell lines, CAOV3 cells had the lowest expression of miR-362-3p while HO-8910 cells had the highest expression. Therefore, CAOV3 and HO-8910 cells were transfected with the miR-362-3p mimic and the miR-362-3p inhibitor, respectively, for further analysis. According to the results shown above, we deduced that miR-362-3p might play a vital role in ovarian cancer.

### miR-362-3p inhibits ovarian cancer cell proliferation and migration

**Fig. 2:**
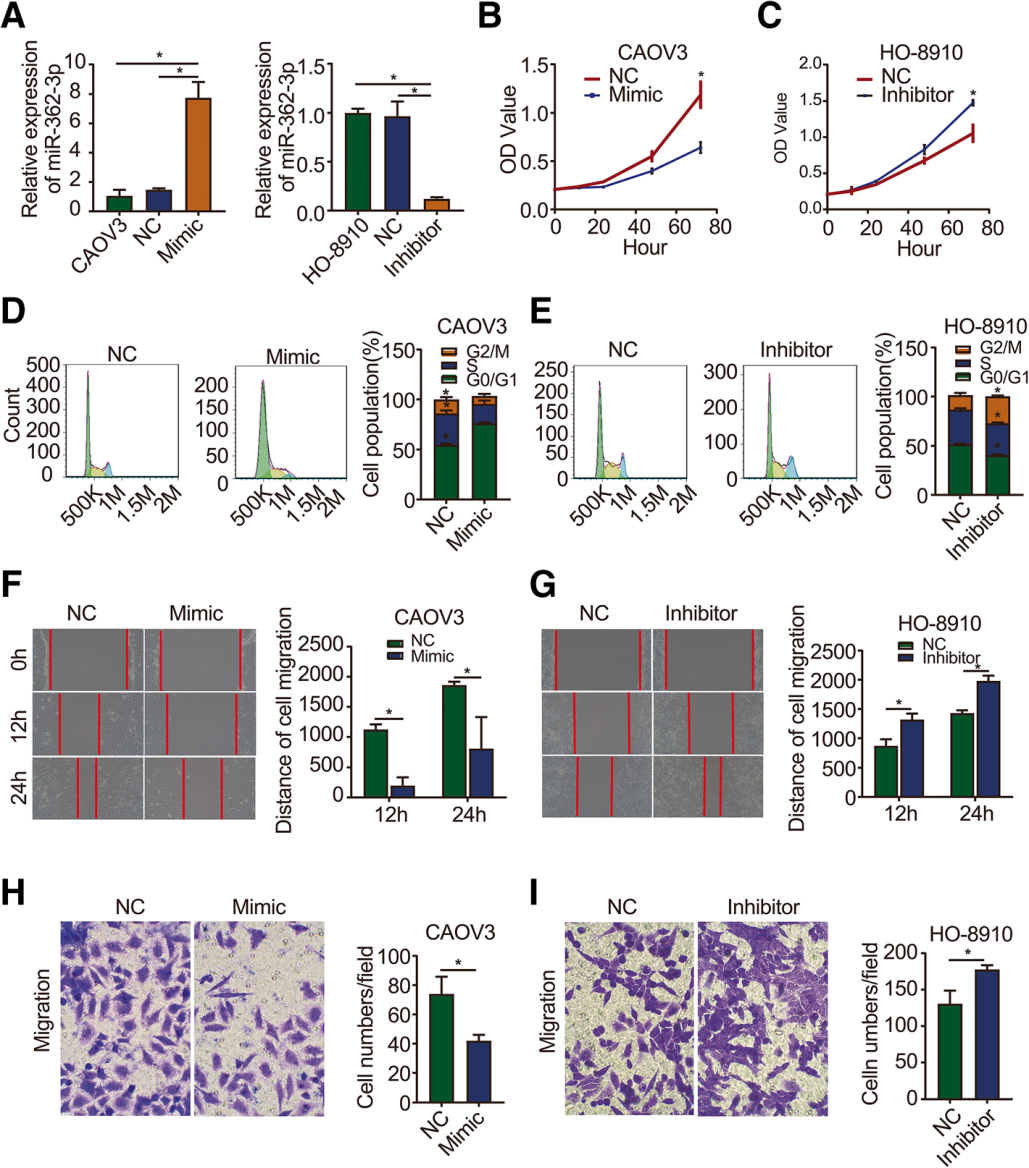
miR-362-3p inhibits ovarian cancer cell proliferation and migration. **a** qPCR analysis of miR-362-3p expression in ovarian cancer cells. CAOV3 was used as a blank control; NC: transfected with a scrambled sequence; mimic or inhibitor: transfected with a miR-362-3p mimic or inhibitor. **b, c** The proliferation of ovarian cancer cells was detected at the indicated time points by CCK-8 assay. **d, e** The proliferation of ovarian cancer cells was detected by a cell cycle assay. **f, g** Wound healing assay of ovarian cancer cells showed that changes in miR-362-3p effectively affected cell motility. **h, i**. Migration assay of ovarian cancer cells showed that changes in miR-362-3p effectively affected cell migration (magnification 200×). Data are shown as the means ± SDs. *P < 0.05

To further evaluate the biological effect of miR-362-3p on ovarian cancer, we upregulated its expression by transfecting SKOV3 cells with the miR-362-3p mimic and downregulated its expression by transfecting HO-8910 cells with the miR-362-3p inhibitor. qRT-PCR was used to detect the silencing and overexpression efficiencies in SKOV3 and HO-8910 cells, respectively. The results showed that miR-362-3p expression was significantly higher in the mimic group and significantly lower in the inhibitor group than in the control group (Fig. 2a). A CCK-8 assay was performed to detect cell proliferation. Cell growth curves showed that the miR-362-3p mimic significantly inhibited the proliferation of CAOV3 cells (Fig. 2b), while the miR-362-3p inhibitor promoted the proliferation of HO-8910 cells (Fig. 2c). Next, a cell cycle analysis was conducted to detect cell proliferation, which revealed that the miR-362-3p mimic significantly decreased the proportion of G2/M phase cells and increased the proportion of G0/G1 phase cells (Fig. 2d). In contrast, the miR-362-3p inhibitor significantly increased the proportion of G2/M phase cells and decreased the proportion of G0/G1 phase cells (Fig. 2e). These data reveal the role of miR-362-3p in the inhibition of cell proliferation.

Wound healing and Transwell migration assays were performed to examine the cell migration ability. The wound-healing assay showed that compared to NC cells, cells transfected with the miR-362-3p mimic exhibited significantly suppressed migration and wound healing (Fig. 2f). On the other hand, the miR-362-3p inhibitor significantly promoted the migration of cells (Fig. 2g). For the migration assay, the number of cells penetrating the lower surface of the membrane in the miR-362-3p mimic group was significantly lower than that in the NC group (Fig. 2h). In contrast, the number of cells penetrating the lower surface in the miR-362-3p inhibitor group was significantly higher than that in the NC group (Fig. 2i).

### SERBP1 is a direct target of miR-362-3p

**Fig. 3:**
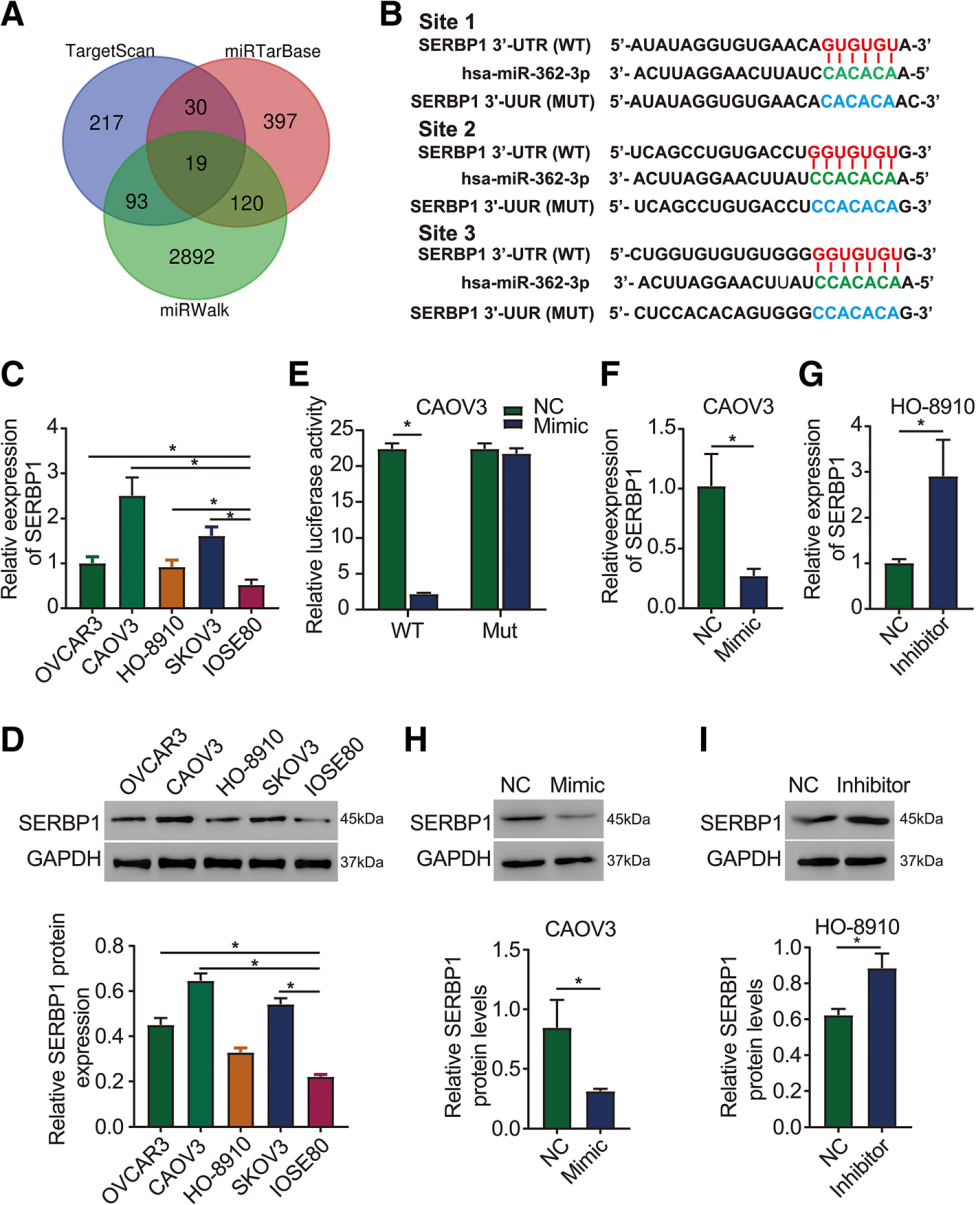
SERBP1 is a direct target of miR-362-3p. **a** Nineteen target genes that can bind to miR-362-3p were selected using three online bioinformatics prediction software programs (TargetScan, miRWalk, and miRTarBase). **b** Putative wild-type (WT) and mutant-type (MUT) miR-362-3p target sequences of SERBP1 mRNA 3’ UTRs. **c, d** SERBP1 mRNA and protein expression in ovarian cancer cell lines are upregulated compared to that in normal ovarian epithelium cell lines. **e** Relative luciferase activity in ovarian cancer cells cotransfected with constructs carrying wild-type or mutant SERBP1 mRNA 3’ UTRs with the miR-362-3p mimic. **f-i** Effects of miR-362-3p mimic and inhibitor on SERBP1 mRNA and protein expression in ovarian cancer cells, respectively. Data are shown as the means ± SDs. *P < 0.05

To clarify the underlying mechanisms of miR-362-3p in ovarian cancer, the potential targets of miR-362-3p were predicted using TargetScan, miRWalk, and miRTarBase. Nineteen potential target genes of miR-362-3p, including SERBP1, were predicted by all three online database tools (Fig. 3a). We focused on SERBP1, and the binding sites of miR-362-3p in the SERBP1 3’ UTR are shown in Fig. 3b. To validate this prediction, we designed both wild-type (WT) and mutant (MUT) 3’UTR sequences of SERBP1 to ensure the direct binding of miR-362-3p to SERBP1. A luciferase activity reporter assay revealed that cotransfection of the miR-362-3p mimic into CAOV3 cells significantly decreased the luciferase activity in cells with a WT 3’ UTR of SERBP1 but not in cells with a MUT 3’ UTR (Fig. 3e). Next, we detected SERBP1 protein and mRNA expression in normal ovarian cell lines (IOSE80) and ovarian cancer cell lines (OVCAR3, CAOV3, HO-8910, and SKOV3) and noticed that the protein and mRNA expression of SERBP1 was significantly lower in IOSE80 cells than in all ovarian cancer cells tested (Fig. 3c and d). This showed a reverse trend when compared to the expression of miR-362-3p. Moreover, we found that SERBP1 protein and mRNA expression were significantly inhibited by the miR-362-3p mimic and were considerably promoted when miR-362-3p was silenced (Fig. 3f g, h, i). Therefore, the findings of this study suggest that miR-362-3p could negatively regulate SERBP1 expression by directly binding to its 3’ UTR.

### SERBP1 overexpression rescues the effects of miR-362-3p on ovarian cancer cell phenotypes

**Fig. 4:**
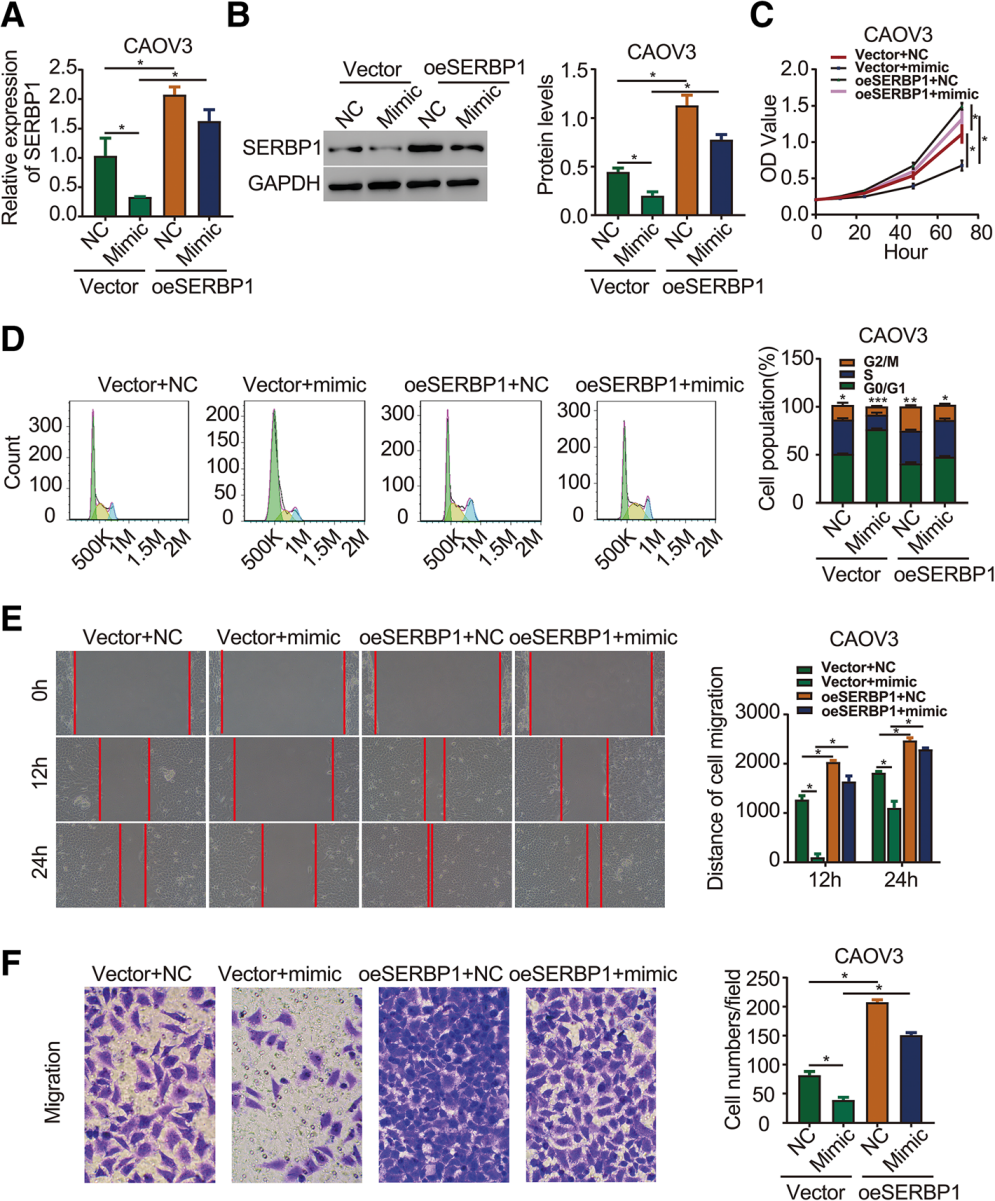
SERBP1 overexpression rescues the effects of miR-362-3p on ovarian cancer cell phenotypes. **a** qPCR analysis showed that overexpression of SERBP1 repressed miR-362-3p mimic-mediated SERBP1 mRNA expression. **b** Western blot analysis showed that overexpression of SERBP1 repressed miR-362-3p mimic-mediated SERBP1 protein expression. **c** CCK-8 assay determined that overexpression of SERBP1 repressed miR-362-3p mimic-mediated CAOV3 cell proliferation. **d** Cell cycle assay determined that overexpression of SERBP1 repressed miR-362-3p mimic-mediated CAOV3 cell proliferation. **e** Wound healing assay determined that overexpression of SERBP1 repressed miR-362-3p mimic-mediated CAOV3 cell migration. **f** Migration assay determined that overexpression of SERBP1 repressed miR-362-3p mimic-mediated CAOV3 cell migration (magnification 200×). oeSERBP1: overexpression of SERBP1. Data are shown as the means ± SDs *P < 0.05

To determine whether SERBP1 is an effector of miR-362-3p in ovarian cancer, we cotransfected the miR-362-3p mimic and pcDNA3.1(+)-SERBP1 (SERBP1 overexpression) vector into CAOV3 cells to conduct rescue experiments. The mRNA and protein levels of SERBP1, shown in Fig. 4a and b, revealed that SERBP1 overexpression significantly increased the SERBP1 mRNA and protein levels that were decreased by the mimic. Moreover, CCK-8 and cell cycle analysis assays showed that the miR-362-3p mimic inhibited ovarian cancer cell proliferation, whereas SERBP1 overexpression significantly reversed this inhibitory effect (Fig. 4c and d). In addition, wound healing and Transwell migration assays showed that SERBP1 overexpression significantly reversed the effect of the miR-362-3p mimic on the migration ability of CAOV3 cells (Fig. 4e and f). These data collectively reveal that miR-362-3p exerts its tumor suppressor effects by targeting SERBP1 expression.

### miR-362-3p suppresses ovarian cancer growth and invasion in vivo

**Fig. 5:**
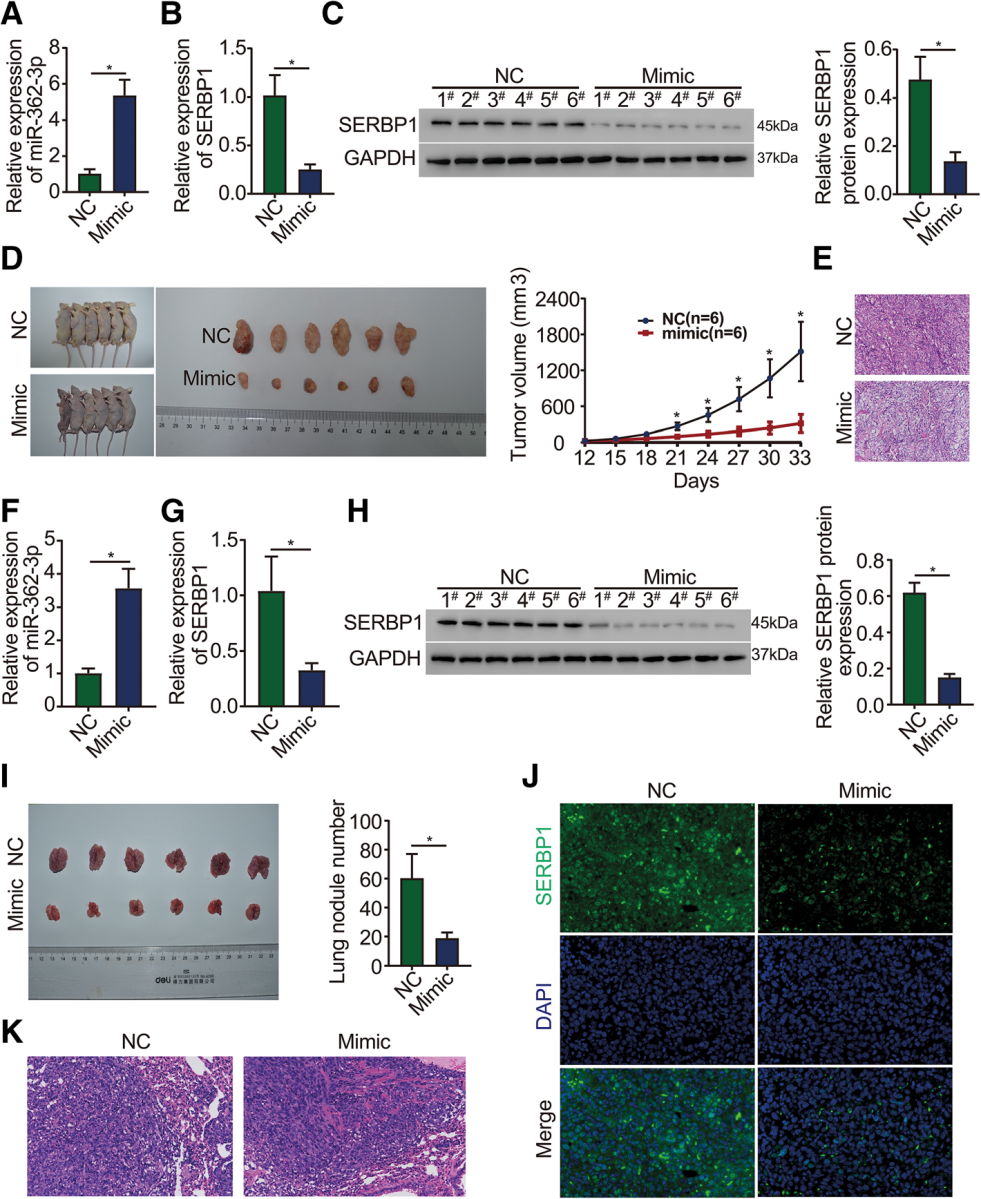
miR-362-3p suppresses ovarian tumor growth and invasion in vivo. **a** qPCR analysis of miR-362-3p expression in tumor tissue from the mimic and NC groups. **b** qPCR analysis of SERBP1 mRNA expression in tumor tissues. **c** Western blot analysis of SERBP1 protein expression in tumor tissues. Each band represents a sample from six individual mice. **d** miR-362-3p mimics inhibited ovarian cancer growth in the xenograft mouse model. **e** Representative images of H&E staining of tumor sections (magnification 200×). (**f**) qPCR analysis of miR-362-3p expression in metastatic lung tumor tissues. **g** qPCR analysis of SERBP1 mRNA expression in metastatic lung tumor tissues. **h** Western blot analysis of SERBP1 protein expression in metastatic lung tumor tissues. Each band represents a sample from six individual mice. **i** The miR-362-3p mimic decreased the number of lung nodules. **j** Representative images of immunofluorescence staining showed that SERBP1 protein expression was downregulated in metastatic lung sections of the mimic group (magnification 400×). **k**. Representative images of H&E staining of metastatic lung sections (magnification 200×). *N* = 6/group for triplicate assays. Data are shown as the means ± SDs. *P < 0.05

To clarify the role of miR-362-3p in vivo, a xenograft model was generated by subcutaneously injecting CAOV3 cells into BALB/c nude mice. Then, the mimic and NC were randomly injected into the tail vein for three weeks. The mRNA level of miR-362-3p in tumor tissues was significantly higher in the mimic group than in the NC group, indicating effective overexpression efficiency (Fig. 5a). In contrast, the mRNA and protein levels of SERBP1 in tumor tissues were significantly lower in the mimic group than in the NC group (Fig. 5b and c). The average tumor volumes in the mimic group were found to be much smaller than those in the NC group (Fig. 5d). Hematoxylin and eosin (H&E) staining was applied to detect changes in tumor cell morphology [14]. H&E analysis showed that necrotic lesions in mimic group tumors were markedly increased compared to that in NC group tumors (Fig. 5e). The data indicate that miR-362-3p can exert a significant inhibitory effect on CAOV3 tumor growth.

To examine the effect of miR-362-3p on ovarian cancer metastasis, CAOV3 cells were injected into the tail veins of BALB/c nude mice. After 6 weeks of injection, the mRNA levels of miR-362-3p in lung tissues were significantly higher in the mimic group than in the NC group (Fig. 5f), and the mRNA and protein levels of SERBP1 were significantly lower in the mimic group than in the NC group (Fig. 5g h). Moreover, mice that had been injected with the mimic showed fewer lung nodules than mice injected with the NC (Fig. 5i). Fluorescence imaging of lung tissues showed that the mice injected with the mimic had weaker green fluorescence signals than the mice injected with the NC (Fig. 5j). H&E staining analysis of lung tissues revealed that necrotic lesions in mimic group tumors were also markedly increased compared to those in NC group tumors (Fig. 5k). These findings indicate that miR-362-3p can exert a significant inhibitory effect on metastasis in vivo.

## Discussion

Despite recent developments in treatment methods for ovarian cancer (including bevacizumab, PARP inhibitors, and platinum), ovarian cancer remains a life-threatening disease with a poor prognosis [15]. A key factor for the poor prognosis of this disease has been our inadequate understanding of the initiating events that induce ovarian cancer and disease progression. Thus, innovative molecular targeting agents are required to overcome this disease.

miRNAs are a type of small noncoding RNA that can posttranscriptionally regulate gene expression, and they function in multiple biological processes. A growing body of evidence has implied that miRNAs are involved in tumorigenesis, acting as either oncogenes [16, 17] or tumor suppressors [18, 19]. More importantly, it has been demonstrated that miRNAs may be potential effective biomarkers for some tumors and thus have implications for use in therapeutic practice. For example, Lin’s study revealed that the oncogene miR-154-5p regulates cellular function and acts as a molecular marker with a poor prognosis in renal carcinoma [20]. Recognizing the key role of miRNAs in the regulation of some diseases, many researchers have focused on the development of miRNA inhibitors. Foinquinos A. and colleagues synthesized an inhibitor of miR-132, which has been considered a target for heart failure therapy, and they revealed the compound’s therapeutic efficacy in various models [21]. Thus, miRNA inhibitors might be another new idea and direction for future tumor treatment.

miR-362-3p has been shown to participate in the regulation of various biological processes, including cell differentiation, apoptosis, proliferation [11, 22], atherosclerosis [22], inflammatory response [23], and diabetes [24]. Furthermore, some studies have reported its pivotal effect on tumors. Assiri et al. [25] revealed that miR-362-3p mediates the transcriptional regulation of the human ether-a-go-go-related gene (hERG) and is associated with survival in breast cancer [25]. Christensen’s study concluded that miR-362-3p may be a novel prognostic marker for colorectal cancer, and this study hypothesizes that the positive effects of augmented miR-362-3p expression may in part be mediated through hypothetical target protein 1 (PTPN1) [26]. Wang et al. illustrated that miR-362-3p functions as a tumor suppressor in renal cell carcinoma and that it might also serve as a potential molecular target in the treatment of renal cell carcinoma [27]. However, the possible role of miR-362-3p in the regulation of the occurrence and development of ovarian cancer is still unknown.

In the present study, the expression and biological roles of miR-362-3p in ovarian cancer were investigated for the first time. Tissue array analysis from the GEO database showed that the expression of miR-362-3p was higher in ovarian cancer tissues than in normal control tissues. Our data are consistent with the bioinformatics analysis of the mRNA expression level of miR-362-3p in ovarian cancer cells, which was lower than that in normal cells. Considering these results, we subsequently took further steps to assess the functional role of miR-362-3p upregulation and downregulation in ovarian cancer development. Through transduction using a miR-362-3p mimic and a miR-362-3p inhibitor, we showed that the miR-362-3p mimic had an anticancer effect by inhibiting the migration and proliferation of ovarian cancer cells. miR-362-3p mimic treatment inhibited cell growth by inducing a decrease in G2/M phase cells and an increase in G0/G1 phase cells in the CAOV3 cell line. In contrast, the miR-362-3p inhibitor showed an oncogenic role in ovarian cancer cells. Subsequently, our data were reinforced by the in vivo experiment, which also supported a tumor-suppressive role for miR-362-3p. Animal experiments demonstrated that miR-362-3p can inhibit the growth of subcutaneous xenografts and decrease the number of nodules in the lung tissues of nude BALB/c mice. Altogether, our data might provide novel insights into the molecular mechanisms underlying the effects of miR-362-3p on ovarian cancer.

Aberrant gene expression plays a crucial role in the occurrence and development of tumors, often followed by a series of downstream target gene alterations and subsequent biological changes. miRNAs exert their biological function by pairing with the 3’ UTRs of target mRNAs [28]. SERBP1 is a plasminogen activator inhibitor 1 mRNA-binding protein encoded by the SERBP1 gene, which is located on chromosome 1p31 [29]. SERBP1 is believed to play a crucial role in various physiological processes, including fibrinolysis, wound healing, and angiogenesis, as well as in tumor cell metastasis and invasion [30, 31]. In addition, Serce et al. [32] found that SERBP1 was abundantly expressed in human breast cancer and may represent a novel breast tumor marker with prognostic significance. More importantly, overexpression of SERBP1 was detected in ovarian cancer and is significantly associated with advanced tumor stages [33]. In this study, by using bioinformatics analysis, we predicted 19 potential target genes of miR-362-3p from all three online database tools, including SERBP1. Due to the pivotal role of SERBP1 in ovarian cancer, we finally decided to verify it as a target gene of miR-362-3p. A luciferase activity reporter assay revealed that miR-362-3p can directly bind to SERBP1. Next, we found that miR-362-3p not only interfered with the expression of the SERBP1 mRNA levels but also significantly suppressed its protein levels. Moreover, the findings of this study revealed that miR-362-3p regulates the expression of SERBP1 in ovarian cancer growth and metastasis. Taken together, our study determined that the function of the miR-362-3p-SERBP1 axis was largely a mechanism to inhibit ovarian cancer occurrence and development. Thus, this detailed elucidation of the mechanisms underlying miR-362-3p activity might supply meaningful information for the future development of ovarian cancer treatments.

In summary, our data reveal that miR-362-3p has an ovarian cancer inhibitory effect. miR-362-3p inhibited the development and progression of ovarian cancer by directly binding to the target gene SERBP1. These data indicate that miR-362-3p might be used as a potential therapeutic agent for ovarian cancer. However, further research may be crucial.

## Data Availability

The analyzed data sets generated during the study are available from the corresponding author on reasonable request.

## Abbreviations

miRNA: microRNA
3’ UTR: 3’ untranslated region
SERBP1: SERPINE1 mRNA binding protein 1
GEO: Gene Expression Omnibus
HSOC: high-grade serous ovarian carcinomas
CCOC: clear cell ovarian carcinomas
OSE: ovarian surface epithelium
FC: fold change
ATCC: American Type Culture Collection
FBS: fetal bovine serum
GAPDH: glyceraldehyde-3-phosphate dehydrogenase
NC: negative control
qRT-PCR: quantitative reverse transcription–polymerase chain reaction
RIPA: radioimmunoprecipitation assay
BSA: bovine serum albumin
SDS–PAGE: sodium dodecyl sulfate polyacrylamide gel electrophoresis
DAPI: 4,6-diamidino-2-phenylindole
